# 2078. Comparison of Complication Types in Patients Receiving Vesicant Intravenous Antimicrobials or Vasopressors via Midlines and PICCs: A Retrospective Review

**DOI:** 10.1093/ofid/ofad500.148

**Published:** 2023-11-27

**Authors:** Bryan V Grigg, Nishant Varghese, Christi L Knapp, Sabra L Shay, Geraldine Jones, James P Herlihy, Prasad Manian, Bradley Lembcke, Mayar Al Mohajer

**Affiliations:** University of Washington, Seattle, WA; Baylor St. Luke's Medical Center, Houston, Texas; Baylor St. Luke's Medical Center, Houston, Texas; Premier Inc, Colorado Springs, Colorado; Baylor St. Luke's Medical Center, Houston, Texas; Baylor College of Medicine, Houston, Texas; Baylor College of Medicine, Houston, Texas; Baylor St Luke's Medical Center, Houston, Texas; Baylor College of Medicine, Houston, Texas

## Abstract

**Background:**

Peripherally inserted central catheters (PICC) are frequently used for long-term intravenous access in hospitalized patients; however, their use is associated with an increased risk of catheter-related bloodstream infection (CRBSI). Midline catheters (MC) have emerged as a potentially safer alternative and are associated with reduced rates of CRBSI but increased risk of thrombosis. Until recently, the use of several vasopressors and antibiotics via MC has been limited due to unknown adverse events. We aimed to compare the hazard ratios for CRBSI, thrombosis, catheter occlusion, and overall adverse events (AE) in adult intensive care unit (ICU) patients receiving vesicant antibiotics or vasopressors through PICC or MC.

**Methods:**

We reviewed patients in 13 ICUs at one quaternary care center from April 2021 to September 2022 who received selected vesicant antibiotics (vancomycin, meropenem, and fluoroquinolones) or vasopressors through either PICC or MC. Patients who received both PICC and MC or other central access during the admission were excluded. Hazard ratios (HR) were compared using Kaplan-Meier survival curves and the log-long rank test. Cox regression was applied to adjust for potential confounding factors, including age, gender, ICU type (medical vs. surgical), duration of therapy, and length of stay.

**Results:**

445 patients (359 MCs, 86 PICCs) were included with 4,897 device days (3,910 MCs, 987 PICCs). Patient characteristics were similar across the two arms (Table 1). Figure 1 shows Kaplan-Meier survival estimate curves comparing PICC and MC. After adjusting for confounders (Table 2), the HR for CRBSI (0.86 [95% CI, 0.08 – 9.41)] and thrombosis (0.41 [95% CI, 0.09- 1.91]) were similar between the two devices. Compared to MC, PICCs were associated with higher occlusion rates (HR, 11.93 [95% CI, 2.97 – 47.89]). There were no significant differences in overall AE between the two groups (HR 1.72 [95% CI, 0.81 – 3.64]).

**Table 1:**
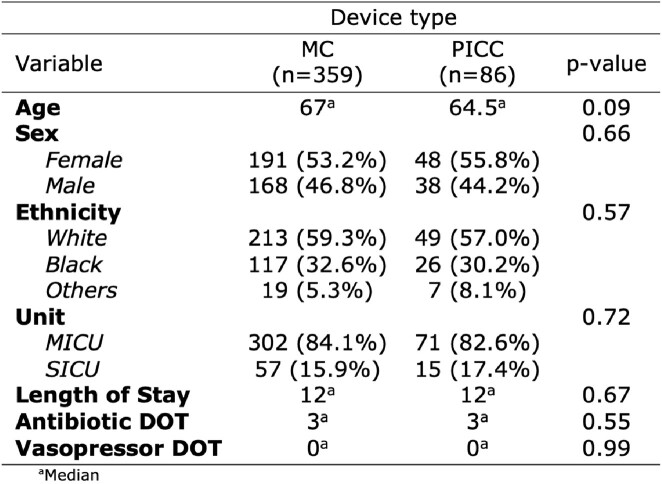
Characteristics of patients who received PICC or MC.

**Figure 1:**
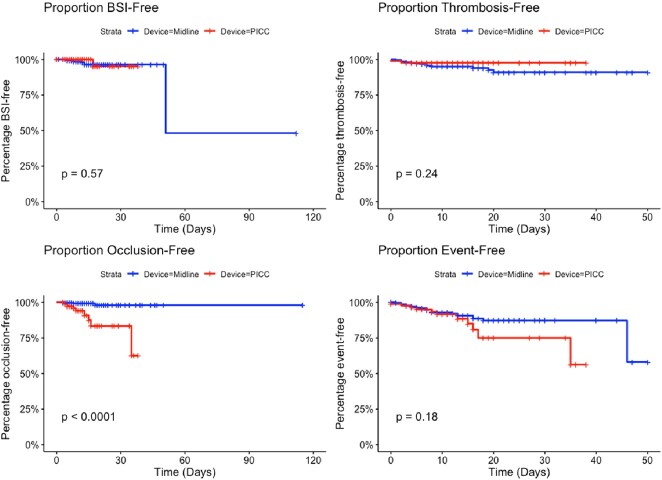
Kaplan-Meier Survival Estimate Curves Comparing PICC and MC. PICC: Peripherally Inserted Central Catheter; MC: Midline Catheter; The log-rank test was used for statistical significance.

**Table 2:**
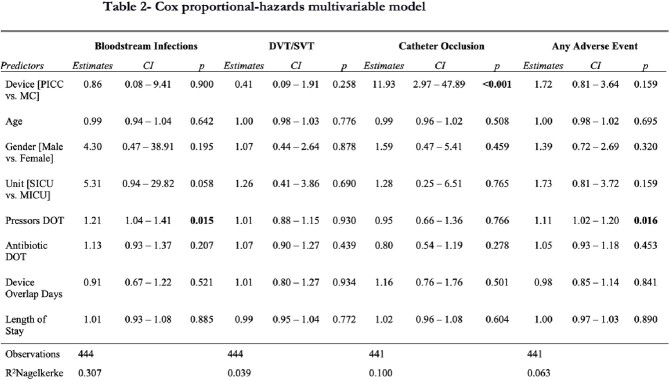
Cox proportional-hazards multivariable model. DVT: Deep Vein Thrombosis; SVT: Superficial Vein Thrombosis; PICC: Peripherally Inserted Central Catheter; MC: Midline Catheter; SICU: Surgical Intensive Care Unit; MICU: Medical Intensive Care Unit; DOT: Days of Therapy; CI: 95% Confidence Interval

**Conclusion:**

This study suggests similar infection and thrombosis hazards in ICU patients receiving vesicant antibiotics and vasopressors via PICC or MC. Notably, the study was not sufficiently powered to detect differences in outcomes. Larger studies are needed to assess the safety of administering these medications in MCs.

**Disclosures:**

**Sabra L. Shay, BSN, MPH**, Premier Inc.: Employee

**Table 1:** Characteristics of patients who received PICC or MC.

MC: Midline Catheter; PICC: Peripherally Inserted Central Catheter; MICU: Medical Intensive Care Unit; SICU: Surgical Intensive Care Unit; DOT: Days of Therapy

